# Explainable AI in kidney stone detection and segmentation: a mini review

**DOI:** 10.3389/fdgth.2026.1750411

**Published:** 2026-05-12

**Authors:** Md Jakir Hossen, B. M. Taslimul Haque, Hasanul Bannah, Md Arifur Rahman, Abir Ahmed, Abdullah Al Noman

**Affiliations:** 1 Center for Advanced Analytics, COE for Artificial Intelligence Faculty of Engineering & Technology, Multimedia University, Melaka, Malaysia; 2 Elite Research Lab, LLC, New York, NY, United States; 3 Information Systems, Central Michigan University, Mount Pleasant, MI, United States; 4 Faculty of AI and Engineering, Multimedia University Cyberjaya, Cyberjaya, Malaysia; 5 College of Graduate and Professional Studies, Trine University, Angola, IN, United States; 6 Department of Information Technology, Washington University of Science & Technology, Alexandria, VA, United States; 7 Wilmington University, New Castle, DE, United States

**Keywords:** clinical decision support, deep learning, explainable AI, grad-CAM, kidney stone segmentation, lime, medical imaging, shap

## Abstract

Kidney stones are one of the most common renal disorders that can produce severe complications if not diagnosed and treated early. Recently, advances in AI have ensured that deep learning and explainable AI enable the automatic segmentation and detection of kidney stones from medical imaging, thus improving diagnostic efficiency and accuracy. For this review, eighteen representative studies using machine learning, deep learning, and hybrid models for kidney stone segmentation were considered, which were published in the period between 2020 and 2025. The XAI techniques being mainly utilized with the discussed models in the study are SHAP, LIME, Grad-CAM, Layer-wise Relevance Propagation, and EigenCAM. Such approaches tend to enhance clinicians’ trust in allowing early diagnosis and supporting clinical decision-making, especially in resource-constrained settings. Regardless of the towering results, this area still suffers due to certain limitations such as lack of diversity in datasets, absence of multimodal integration, and scarcity of real-world validation. All in all, integrating DL with XAI presents a transparent, reliable, and clinically acceptable approach to detecting and segmenting kidney stones.

## Introduction

Kidney stone disorders are the most common urinary tract disorders; if untreated, they can lead to severe pain, infections, and kidney damage in millions of people throughout the world. It hence becomes very important to carry out the detection and segmentation of kidney stones based on medical imagery for appropriate intervention. Most traditional diagnostic approaches depend upon the interpretation by radiologists based on CT scans, x-rays, or ultrasound images, which may be time-consuming and prone to variability.

Recent advances in deep learning have transformed the landscape of medical image analysis. Segmentation of kidney stones can nowadays be automatically and highly accurately performed. However, the clinical translation of many of these deep learning models remains limited because the majority of them are inherently “black-box.” Transparency and interpretability of clinical decisions require clinicians to trust AI-assisted decisions. Explainable AI techniques provide insights into model predictions by highlighting critical regions within the medical images themselves. Several techniques, such as Grad-CAM, SHAP, LIME, Layer-wise Relevance Propagation, and EigenCAM, make it possible for clinicians to comprehend and visualize the way AI models identify kidney stones; thus, providing the much-needed confidence in AI-assisted diagnosis.

Table 1 summarizes eighteen recent works on integrating XAI with deep learning models applied to the segmentation of kidney stones. Although this review emphasizes segmentation, it is important to note that several included studies focus on related tasks such as detection, classification, chronic kidney disease prediction, and urinalysis. Works like Bhandari et al. (2), Sharon et al. (18), and Begum et al. (3) rely on CNN-based or Vision Transformer-based architectures combined with Grad-CAM or SHAP for spatial- and feature-level explanations. Other recent related studies include, for example, Pradhan et al. (1) and Jawad et al. (15) that use SHAP and LIME in order to provide insight into the clinical features of the formation of the stones. These studies indicate that XAI enhances model transparency and improves clinical decision-making and early detection, which can be deployable even in resource-constrained environments.

**Table 1:** Summary of representative studies on kidney stone segmentation using explainable AI (XAI).

Citation	Dataset	Method	Used XAI	Application of used XAI	Purpose in Healthcare	Limitations
Pradhan et al. (1)	Six clinical feature—gravity, pH, osmolality, conductivity, urea, and calcium—along with a target variable which indicates the presence or absence of kidney stone.	Machine Learning based predictive framework.	SHAP (SHapley Additive exPlanations) and LIME (Local Interpretable Model-agnostic Explanations).	SHAP and LIME was used to analyze feature importance. SHAP (SHap- ley Additive exPlanations) showed that calcium, osmolality, and urea levels were the most significant contributors to kidney stone predictions.	Suggests their role in assisting physicians with diagnosis, especially in low-resource environments where imaging costs can be prohibitive.	Limited clinical and lifestyle variables and not use biomarkers or metabolic parameters.
Bhandari et al. (2)	Data collected from multiple hospitals in Dhaka, Bangladesh (3,709 cysts; 5,077 normal; 1,377 stones; 2,283 tumors).	CNN model and an XAI-based explanation framework.	SHAP (SHapley Additive exPlanations) and LIME (Local Interpretable Model-agnostic Explanations).	SHAP assessed the influence of the model features. LIME image explainer was used to extract numerical features and perturbations.	The proposed methodology could assist medical teams in making more accurate diagnoses and treatments.	Limited number of CT samples, not apply data augmentation and not combining DL models with other XAI.
Begum et al. (3)	Kidney CT and clinical metadata	Combines Vision Transformers (ViT), structured tabular data encoders, multimodal fusion, and privacy-preserving federated learning. In addition, SHAP and Grad-CAM are used to ensure interpreted results.	SHAP (SHapley Additive exPlanations) and Grad-CAM (Gradient-weighted Class Activation Mapping)	SHAP analysis revealed clinically significant factors. Grad-CAM heat maps confirmed the exact localization of the attention on the functioning of relevant kidney regions.	Keeping the data confined in each of the respective nodes, ensuring strict data privacy, a major requirement within modern healthcare environments.	Model is limited to kidney stone detection, lacks multi-label classification and integration of additional clinical data, and has not been validated for real-time deployment or diverse populations.
Islam et at. (4)	CT KIDNEY DATASET: Normal-Cyst-Tumor and Stone.	six models to investigate data, including three Visual Trans former variants (EANet, CCT, and Swin Transformer), Inception v3, and Vgg16 and Resnet 50.	Grad-CAM (Gradient-weighted Class Activation Mapping)	Grad-CAM analysis of kidney Cyst, Normal, Stone and Tumor class photos at the final convolution layer in the Inception v3, Vgg16, and Resnet models.	Providing explainable AI outputs to increase clinician trust in AI-assisted diagnosis. Facilitating low-cost, efficient diagnostics, especially in resource-limited clinical settings.	Limited number of CT samples, Data augmentation was not applied, Integration of additional clinical data (like lab results, patient history) was not included.
Jimenez et al. (5)	Ex-vivo kidney stone image dataset.	Causal Explanation Score (CaES) and using this validate the outputs of a DL model.	Grad-CAM (Gradient-weighted Class Activation Mapping).	Grad-CAM maps indicate the most relevant area in the input image for its corresponding classification	Enabling healthcare specialists to leverage DL model findings for diagnosis, grasping the logic behind such results	CaES doesn’t handle confounded variable relationships, hasn’t been tested across different CNNs, small dataset and lower classification performance may also affect results.
Ahmed et al. (6)	Data collected from a MAYO hospital in Lahore, Pakistan- KUB x-ray images	VGG16 CNN with transfer learning on augmented KUB x-ray images, combined with Layer-Wise Relevance Propagation (LRP) for explainable kidney stone detection.	Layer-wise relevance propagation.	Clarify the reasoning behind the model's predictions, thereby promoting transparency and fairness in the kidney stone identification process.	This approach provides a transparent and effective solution for arriving at definitive diagnostic conclusions, reducing the time needed for diagnosis and enhancing diagnostic accuracy.	One of the critical limitations is the availability of high-quality and diverse medical image data of KUB x-rays of kidney stones.
Bayram et al. (7)	Data collected from multiple hospitals in Dhaka, Bangladesh. The data set includes patients diagnosed with namely kidney cyst, normal and stone findings.	YOLOv7 object-detection architecture on CT images (kidney, cyst and stone classes) augmented with explainable AI (xAI) features.	Grad-CAM (Gradient-weighted Class Activation Mapping).	Prove the performance of the outputs produced by the YOLOv7 architecture design and to make it more clear and reliable by producing visual explanations for AI model.	The AI assisted diagnosis system has been developed, with the use of developed system can be reduce the workload of health employees.	Small, localized dataset, class imbalance, focus only on stones and cysts, use of 2D CT images, lack of clinical validation, limited explainability.
Shikdar et al. (8)	9,416 ultrasound images of two class: kidney stones and normal from Kaggle.	Deployed four pretrained deep learning models, which are DenseNet201, ResNet50, InceptionV3, and MobileNetV3. Methods like MixUp and CutMix, known as augmented regularization, were used to make the model more robust.	Saliency map Explainable AI	Highlights the region that are responsible for the model prediction. A heatmap is being generated that highlights the affected region making it easier for an expert to double check the diagnosis.	Support clinical decision-making using accurate tools like AI-assisted ultrasound analysis.	A two-class dataset that may miss rare anomalies, reliance on 2D ultrasound images, lack of external clinical validation, use of only saliency maps for explainability,
Dillibabu et al. (9)	CT scan images (Cyst, Normal, Stone, Tumor) from grayscale CT slices obtained from open-source and clinical repositories,	CNN-LSTM model for multiclass classification of kidney diseases from CT scan images.	Grad-CAM (Gradient-weighted Class Activation Mapping).	To ensure transparency and explainability in model decision-making.	Kidney diseases such as stones and tumors often exhibit overlapping characteristics in imaging. Early and accurate detection can lead to timely interventions, reduce diagnostic burden on radiologists, and improve patient outcomes.	The dataset, though balanced and diverse, is not extensive enough to capture rare anomalies.
Batra et al. (10)	CT images acquired from the Picture Archiving and Communication System (PACS) of various hospitals in Dhaka, Bangladesh.	Deep learning within a federated learning paradigm.	Grad-CAM (Gradient-weighted Class Activation Mapping).	For highlighting the discriminative regions that the central model focused on when making predictions across different classes in the AD–D dataset.	Provide valuable insights into the framework's adaptability and robustness in real-world clinical settings with diverse and challenging data distributions.	Focus on classification only, use of limited and non-diverse datasets, evaluation with few clients, reliance on a single XAI method (Grad-CAM), and lack of real-world clinical validation.
Naznine et al. (11)	Urine Sediment Dataset (USE) and an independent clinical microscopy dataset collected from symptomatic UTI patients at a specialist LUTS outpatient clinic in central London.	An ensemble approach combining YOLOv9e and KD-YOLOX-ViT was used, with predictions aggregated via Weighted Box Fusion (WBF). EigenCAM was applied for model interpretability.	EigenCAM (Class Activation Mapping) Explainable Artificial Intelligence.	EigenCAM is employed to provide interpretability to the model's predictions. EigenCAM identifies and emphasizes the specific areas in the input images that have a major influence on the decision-making process of the model.	The adoption of such technologies in pediatric urology and broader urinalysis could greatly reduce the workload of healthcare professionals, expedite diagnostic processes, and ensure timely medical intervention.	Slightly lower accuracy compared to top models, limited dataset
Lopez-Tiro et al. (12)	181 kidney stone images.	**Six shallow machine-learning classifiers** and **three deep-learning architectures** to compare their kidney-stone recognition performance.	Grad-CAM (Gradient-weighted Class Activation Mapping).	To Determine the information (i.e., for finding the important color or texture features, or locating the image areas including the most important information) which favour a successful kidney stone recognition.	Fast, accurate, and automated identification of kidney stone types.	The lack of data.
Arifuzzaman et al. (13)	“CT KID- NEY DATASET: Normal-Cyst-Tumor and Stone” collected from various hospitals in Dhaka, Bangladesh.	Ensemble of transfer learning models (EfficientNetV2, InceptionNetV2, MobileNetV2) and Vision Transformer (ViT) to achieve 96% accuracy in early CKD detection.	Local Interpretable Model-agnostic Explanations (LIME)	Detect and emphasize areas in medical images that had a significant effect on the decision-making process of the model. This provided crucial insights into the primary features that drove the model's predictions.	The purpose of this paper in medical care is to develop a fast, accurate, and automated system for diagnosing kidney disease using an ensemble of deep learning models with explainable AI.	Dataset dependence and diversity, clinical validation missing, limited scope of diseases.
Day et al. (14)	*Fourier Transform Infrared (FTIR) spectroscopy* readings of kidney stone samples.	It was tested whether adding AI algorithm overreads to FTIR spectra could improve the detection rate of technologist-misclassified FTIR spectra.	SHAP (SHapley Additive exPlanations)	SHAP analysis was performed to generate predictions across a range of correct and incorrect predictions.	Improve accuracy of kidney stone identification, reduce human error, support correct treatment decisions.	A major limitation of the quality assurance program was that the manner in which the AI results were used by the practice changed over time.
Jawad et al. (15)	Dataset is collected from UCI Machine Learning Repository on Chronic Kidney Diseases from Apollo Hospital, India.	Missing data analysis by JALittle's MCAR Test, Imputation by ML models and nephrologists, Feature and Example based XAI applications.	SHAP (SHapley Additive exPlanations) and LIME (Local Interpretable Model-agnostic Explanations).	Validated the feature contributions, inter-feature dependence, feature values in CKD vs Non-CKD predictions.	Early CKD prediction, explainable decision-making, clinician support, patient awareness.	Presence of a significant portion of missing values in a sensitive domain like CKD is also a great limitation.
Reddy et al. (16)	12,446 DICOM images—normal (5,077 images), cyst (3,709 images), stone (1,377 images), tumor (2,283 images).		SHAP (SHapley Additive exPlanations) and LIME (Local Interpretable Model-agnostic Explanations).	Highlight important image regions, making model decisions transparent.	Supports early detection of kidney abnormalities, reduces radiologist workload, and improves diagnostic consistency.	Small/single-center dataset, and no real clinical validation reduce the model's generalizability.
Almuayqil et al. (17)	CT kidney dataset collected from hospitals in Dhaka, Bangladesh.	six-phase CNN-based KidneyNet model.	Grad-CAM (Gradient-weighted Class Activation Mapping).	Detects vascular thickening and analyzes the peripheral and diffuse distributions of opacities in MRI images.	The primary goal of this model is to simplify the early detection of CKDs by healthcare professionals, aiming to reduce the time and cost associated with diagnosis.	Geographically restricted Dhaka-based dataset and the computational speed of the KidneyNet model.
Sharon et al. (18)	CT kidney dataset collected from hospitals in Bangladesh.	DBAR_Net model, which is an enhanced convolutional neural network.	SHAP (SHapley Additive exPlanations) and Grad-CAM (Gradient-weighted Class Activation Mapping)	Grad-CAM18 highlights important regions in images that significantly impact the model's predictions for every class. SHAP visualisation offer visual representation that indicate the importance of specific regions on the model's decisions, enhancing its interpretability.	The suggested model assists medical examiners in making more accurate clinical decisions when diagnosing renal diseases	Limited dataset diversity, lack of clinical validation, restricted to CT imaging.

While significant advancements have been made, a number of these challenges include limited diversity in datasets, very much restricted clinical validation, most using single XAI techniques, and a lack of adequate multi-modal data integration. Overcoming such limitations is required to ensure that the deployment of XAI-driven kidney stone segmentation systems is safe, effective, and scaled. Therefore, this review covers the DL-XAI integrated approach for an accurate, interpretable, clinically trustworthy method of detecting kidney stones.

## Methodology of literature search

A structured mini-review approach was adopted to enhance transparency and reproducibility. Literature published between 2020 and 2025 was retrieved from major academic databases, including Google Scholar, IEEE Xplore, ScienceDirect, Springer, Wiley, and MDPI. The keywords involved in this search are “kidney stone segmentation,” “kidney stone,” “explainable AI (XAI),” “Grad-CAM,” “SHAP,” “LIME,” and “chronic kidney disease”.

**Figure 1:**
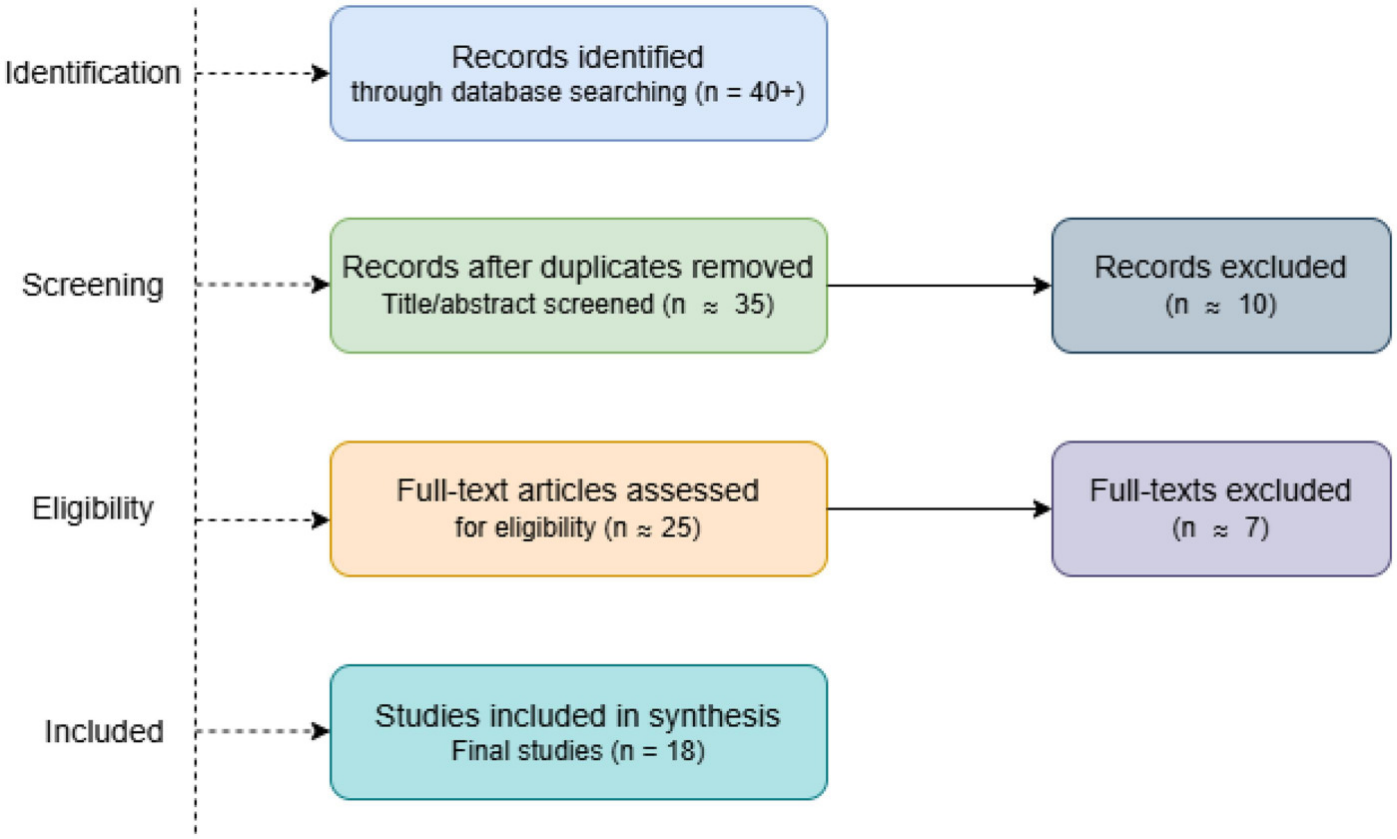
PRISMA flow diagram illustrating the study selection process.

The study selection process was guided by the PRISMA (Preferred Reporting Items for Systematic Reviews and Meta-Analyses) framework to improve clarity in reporting. A PRISMA flow diagram (Figure 1) illustrates the identification, screening, eligibility, and inclusion stages.

Inclusion criteria were defined as:

- peer-reviewed journal articles and reputable conference papers,
- studies applying machine learning or deep learning techniques to kidney stone analysis or related renal imaging tasks,
- explicit use of explainable AI methods,
- publications in English between 2020 and 2025.

Exclusion criteria included:

- non-peer-reviewed articles or opinion papers,
- studies without AI-based methodologies,
- works lacking explainability components,
- duplicate or redundant studies.

These selected studies do not aim at providing an exhaustive sample but illustrate the wide variety of methodologies, approaches to XAI, clinical applications, and current limitations in the context of kidney stone detection and segmentation.

## Comparative analysis and discussion

Table 1 summarizes recent studies involving eighteen applications of XAI techniques in the task of kidney stone segmentation. Approaches include several machine learning and deep learning methods, various medical imaging datasets, and different XAI techniques such as SHAP, LIME, Grad-CAM, Layer-wise Relevance Propagation, and EigenCAM. Of these studies, medical imaging, especially CT and ultrasound scans, emerges as the main application domain due to the important role of image-based diagnosis when it comes to the detection of kidney stones and in the assessment of renal anomalies.

Outstanding among these is the fact that advanced deep learning architectures are combined with interpretability frameworks. Works include Bhandari et al. (2), Arifuzzaman et al. (13), and Sharon et al. (18), which have combined CNN and ensemble models with Grad-CAM or SHAP to highlight regions of interest and hence visually explain model predictions. Similarly, Vision Transformers coupled with SHAP and Grad-CAM, as in Begum et al. (3), show the feasibility of fusing image and tabular clinical data in a privacy-preserving, federated learning setup. Hence, interpretable and distributed prediction can be enabled without collecting data into a single repository.

XAI will play a multifunctional role in these studies. Feature importance quantification is often found through SHAP and LIME among structured clinical data by (1, 15), while Grad-CAM and EigenCAM by (4, 11) are used to localize discriminative regions in imaging datasets. This has therefore increased the trust among clinicians through model transparency by facilitating early detection, improving diagnostic confidence, and supporting low-resource clinical environments where the costs of imaging or dataset availability are limited. In clinical settings, these benefits are most relevant for radiologists (image interpretation), urologists (preoperative planning), and decision support in low-resource environments. However, most studies remain experimental, with limited validation in real clinical workflows or assessment of impact on diagnostic outcomes.

Despite the widespread use of XAI techniques, their comparative effectiveness in kidney stone segmentation remains unclear. Grad-CAM is popular in imaging research for intuitive visualization purposes and for coarse localization, making it less useful for accurate segmentation. In contrast, SHAP and LIME provide good feature-level interpretability for structured data, but are limited in their direct use for image-based segmentation. LRP is able to provide more theoretically based explanations but is less commonly used because of computational complexity. Similarly, EigenCAM and saliency-based approaches increase visualization but may not be consistent and clinically reliable. Most research uses a single XAI technique without benchmarking or validation against ground truth segmentation or expert annotations and raise questions about its clinical applicability. Overall, there is no single optimal method of XAI, and a combination of different techniques might offer more thorough and reliable interpretability, although that has not been extensively explored.

Despite these developments, key limitations persist. Most studies rely on small, geographically restricted datasets, limiting generalizability, and lack integration of diverse clinical variables. Additionally, limited use of multiple XAI methods and insufficient validation across populations or real-world settings reduce their clinical applicability.

By contrast, approaches that combine multiple XAI methods with ensemble or multi-modal models provide more robust actionable insights. Indeed, Begum et al. (3) and Sharon et al. (18) employed combined Grad-CAM and SHAP frameworks to ensure interpretability at the spatial and feature levels, respectively. Similarly, in illustrating temporal dependencies among the CT images in their study, Dillibabu et al. (9) utilized a CNN-LSTM model with the help of Grad-CAM, showcasing that multilayered integration of XAI increases both diagnostic accuracy and explanation fidelity.

This comparative analysis emphasizes three major near-term benefits of using XAI in kidney stone segmentation.

Improved interpretability: Enabling the clinicians to visualize and provide meaning to the model predictions instills more trust in AI-assisted diagnosis.

Automation of expert-intensive tasks: It automates expert-intensive tasks, accelerating the process of the detection, segmentation, and risk stratification of stones, which at the present time are all tasks highly dependent on the workload of radiologists.

Insight Generation: The identification of key features and regions aids in early detection, preventive care, and personalized treatment planning.

Realizing these benefits, however, depends on addressing some persistent limitations- dataset diversity, multimodal integration, multi-XAI frameworks, and real-world clinical validation. Bias audits, cross-center validation, and hybrid approaches to XAI will be important for translation of these promising models into effective clinical tools in the diagnosis and management of kidney stones.

## Conclusion

Explainable AI has emerged as an essential element in the advancement of kidney stone segmentation, addressing the challenge of transparency that has long been observed in deep learning–based medical imaging systems. Traditional deep learning methods perform segmentation with high accuracy, but their black-box nature makes them clinically unacceptable. Furthermore, XAI methods themselves are not without risk — unfaithful explanations or misleading heatmaps, if unvalidated against clinical ground truth, could inadvertently reinforce diagnostic errors rather than prevent them. The integration of XAI techniques, such as Grad-CAM, SHAP, LIME, LRP, and EigenCAM, will appropriately enable clinicians to visualize important image regions, understand feature contributions, and provide insights into model behavior in a medically meaningful way. Thus, XAI shows potential for radiologists at the image-reporting stage and nephrologists during risk stratification, though current evidence remains conceptual and prospective clinical validation is still lacking.

The review of the state-of-the-art is done, concluding that a combination of various XAI methods using an advanced architecture, such as CNN, Vision Transformers, LSTM-based models, and multimodal frameworks, resulted in more insights with improved explanation fidelity. Nevertheless, most of the identified works suffer from deficiencies in at least one of the following: limited diversity of datasets used, general lack of cross-center validation, and reliance on a single XAI technique. Moreover, the limited use of imaging data in a multimodal approach, meaning a combination with clinical or laboratory data, restricts the full potential of XAI-driven diagnostic systems.

Future studies will need to ensure that XAI is implemented in a clinical setting through the development and incorporation of various datasets, multiple-XAI frameworks, strong external validation, and real-world deployment studies. Hybrid AI–XAI approaches can support the development of reliable, transparent, and clinically effective systems for kidney stone segmentation. The integration of advanced XAI techniques holds strong potential for ensuring safe, interpretable, and trustworthy AI solutions in kidney stone diagnosis and management.
